# Digital health interventions for oncofertility in female patients: a systematic review

**DOI:** 10.4069/whn.2025.06.13

**Published:** 2025-06-30

**Authors:** Juyoung Ha, Minji Kim, Hyojin Park

**Affiliations:** 1 College of Nursing, Pusan National University, Yangsan, Korea; 2 Department of Nursing, Catholic University of Pusan, Busan, Korea

**Keywords:** Digital health, Female, Fertility preservation, Neoplasms, Systematic review

## Abstract

**Purpose:**

The importance of fertility preservation during cancer treatment is increasingly emphasized, and the provision of oncofertility care has gained significant attention. This study aims to systematically collect and analyze research on digital interventions related to oncofertility for cancer patients and survivors.

**Methods:**

Following PRISMA 2020 guidelines, a systematic search for studies on digital interventions for oncofertility targeting cancer patients and survivors, published up to November 5, 2024, was conducted using PubMed, Embase, CINAHL, Cochrane Library, and RISS. The retrieved articles underwent screening based on their titles, abstracts, and full texts, and were subsequently selected according to predefined inclusion and exclusion criteria. The quality of the selected studies was assessed using the Risk of Bias 2.0 tool for randomized controlled trials and the Risk of Bias in Non-randomized Studies tool.

**Results:**

From 17,820 retrieved articles, five studies were ultimately selected. Of these, four targeted cancer patients, and one involved cancer survivors. The most common type of intervention was web-based. The studies assessed outcomes across domains including symptom management, emotional, and cognitive functioning, and usability. Significant effects were noted in the emotional domain for fertility-related stress and in the cognitive domain for knowledge.

**Conclusion:**

This review highlights the increasing use of digital health interventions in oncofertility care, with most being web-based. The findings suggest that such interventions may help improve emotional well-being and fertility-related knowledge. Further research is warranted to diversify digital modalities and to develop personalized, evidence-based approaches tailored to the needs of cancer patients.

## Introduction

With the increasing number of young cancer patients and improved survival rates after cancer treatment, fertility preservation during treatment has become an important medical issue that directly affects the quality of life after survival [1]. The term “oncofertility” refers to fertility preservation for cancer patients and is a multidisciplinary approach designed to support the preservation of fertility and future family planning for patients of reproductive age. This involves raising awareness of the risk of impaired reproductive function due to cancer treatment and providing counseling and fertility preservation treatment [2,3]. It is an integrated concept that considers patients’ physical health, emotional stability, cognitive understanding, and decision-making regarding their future plans [1,4]. In particular, fertility is a core element for future planning and psychological stability among young cancer patients, making fertility preservation an essential medical service that should be considered at the start of cancer treatment [2,3].

During cancer treatment, patients experience various oncofertility-related issues, including reduced reproductive function and associated physical changes, the loss of fertility preservation opportunities due to urgent treatment needs, anxiety about forming a family, and uncertainty regarding the possibility of pregnancy after treatment [4,5]. Medical staff support patients’ understanding and decision-making around fertility preservation through the use of visual aids, counseling about preservation methods, and decision support tools [6,7]. However, existing face-to-face oncofertility interventions face challenges in providing individualized support due to time and space limitations and restricted access to healthcare professionals [7]. Patients are increasingly demanding more personalized and repeatedly accessible interventions tailored to their health status, reproductive goals, and personal values [7,8]. In response, digital healthcare technology is being recognized as a promising alternative.

Digital health is defined as a personalized health management approach that uses information and communication technologies—such as web-based programs, mobile applications, virtual reality systems, and wearable devices—to deliver health information, treatment decision support, emotional support, and symptom monitoring [9,10]. Digital health is generally divided into four categories: telehealth, mobile health, health analytics, and digitized health systems [11]. These systems integrate and analyze health information, biological rhythms, behaviors, and daily records collected via personal devices and apps [9,10]. Digital health can enhance patients’ autonomy and healthcare equity before and after treatment [12], while also offering advantages in intervention content and delivery, such as speed, interactivity, and ease of access [9,12].

However, research on digital health interventions for oncofertility remains very limited, with most studies focusing on conceptual definitions, status surveys, or qualitative exploration [6,8]. Moreover, the heterogeneity of intervention methods and outcome assessment metrics creates obstacles to comprehensive interpretation of individual studies [5-7]. In this context, conducting a systematic literature review is crucial to address this heterogeneity and synthesize dispersed findings. Systematic reviews facilitate the comparison and integration of intervention content, evaluation time points, and outcomes across individual studies, thereby establishing a foundation for the development and clinical implementation of advanced interventions [13,14].

Therefore, this study systematically reviews previous studies on digital health interventions for fertility preservation among female cancer patients. The aim is to identify trends in intervention types, structures, primary effect domains, and delivery methods, and to provide an empirical foundation for the future development and practical application of effective interventions.

This systematic literature review was conducted to identify studies on digital health interventions for oncofertility and to evaluate the efficacy of these interventions. The specific objectives were as follows: First, to identify the characteristics of research on digital health interventions for oncofertility. Second, to identify the characteristics of the digital health interventions for oncofertility. Third, to comprehensively review the efficacy of digital health interventions for oncofertility.

## Methods

### Study design

This study is a systematic literature review conducted to identify studies on digital health interventions for oncofertility and to confirm the efficacy of these interventions.

The literature review process was described in accordance with the systematic review reporting guidelines of the PRISMA (Preferred Reporting Items for Systematic Reviews and Meta-Analyses) 2020 [13] and the Cochrane Handbook for Systematic Reviews of Interventions version 6.3 [14].

### Key questions

We conducted our database search and literature review using the participants, intervention, comparison, outcomes, study design (PICO-SD) framework for systematic literature reviews. The study participants (P) were cancer patients or cancer survivors. There were no restrictions on cancer type, but studies focusing exclusively on male participants were excluded. The interventions (I) were digital healthcare-based interventions related to oncofertility; studies that did not fit the definition of digital healthcare, or that did not clearly describe the intervention, were excluded. The control group (C) was not specifically defined or limited in this review. Outcomes (O) included any study that reported at least one outcome variable measured in cancer patients or survivors following digital health interventions for oncofertility. Study design (SD) was limited to randomized controlled trials (RCTs) and nonrandomized experimental studies to confirm the types and efficacy of interventions.

### Literature search strategy

The literature search was conducted without any restriction on publication year, including all studies up to November 5, 2024. Articles published in both domestic and international journals were identified using keywords that combined MeSH and EMTREE-controlled vocabulary with natural language synonyms. International databases searched included PubMed, Embase, CINAHL, and the Cochrane Library, while the domestic database was the Research Information Sharing Service (RISS). For international literature, search terms included (1) oncofertility OR fertility, (2) cancer, and (3) mobile OR web OR online OR app* OR AI OR tele* OR digital OR technology. For domestic literature, similar terms were used in Korean: (1) 온코퍼틸리티 OR 가임력 OR 난임 OR 불임, (2) 암, (3) 모바일 OR 온라인 OR 앱 OR 어플리케이션 OR AI OR 디지털.

### Study selection and data collection

The inclusion criteria were: (1) studies involving cancer patients or survivors, (2) studies applying digital healthcare-based interventions, (3) studies published in English or Korean in academic journals, and (4) studies for which the full text was available.

The exclusion criteria were: (1) studies that did not include female cancer patients or focused only on healthcare professionals, (2) interventions not based on digital healthcare, (3) studies lacking a clear description of the digital health intervention for oncofertility, (4) studies published in languages other than English or Korean, (5) qualitative studies (case reports, descriptive interview studies), methodological studies, mixed-methods studies, protocol studies, meta-analyses, literature reviews, animal experiments, (6) unpublished theses, and (7) studies with unavailable full texts.

Articles identified through electronic databases were systematically organized using Microsoft Excel 2016 (Microsoft Corp., Redmond, WA, USA). After removing duplicates, titles and abstracts were screened according to the selection criteria. The processes of selection and exclusion were documented step by step. All study selection and data extraction were independently conducted by three researchers, with disagreements resolved through discussion.

As digital health interventions for oncofertility remain in the early stages of research, the number of studies meeting the criteria was limited. Therefore, preliminary studies that reported specific early results—such as user response, adherence rates, or acceptability—were included if the intervention had been actually applied to patients [14]. However, studies at the design stage before implementation, or those lacking clear intervention content or results, were excluded.

### Assessment of study quality

The quality of included studies was assessed using Cochrane’s tools: for RCTs, the Risk of Bias 2.0 (RoB 2.0) tool, and for preliminary studies, the Risk of Bias in Non-randomized Studies of Interventions (RoBINS-I) tool [14]. Quality assessment was independently performed by the researchers, with final judgments reached through consensus.

RoB 2.0 evaluates five domains: randomization process, deviations from intended interventions, missing outcome data, measurement of the outcome, and selection of the reported result. Studies were rated as having “low risk of bias” if all domains were low risk, “high risk of bias” if at least one domain was high risk, or “some concerns” if problems were found in at least one domain but the risk was not high.

The ROBINS-I tool evaluates seven domains of bias: bias due to confounding, selection of participants, classification of interventions, deviations from intended interventions, missing data, measurement of outcomes, and selection of the reported result. Results are categorized as “low risk of bias,” “moderate risk of bias,” “serious risk of bias,” “critical risk of bias,” or “no information” if evidence is insufficient to assess the risk of bias.

### Data extraction

Selected studies were systematically organized in Microsoft Excel 2016 according to the PICO-SD criteria. Data extraction focused on the author, publication year, country, sample size, intervention characteristics, and major findings. Because the included studies varied in outcome types and measurement methods, and some did not report statistical values such as means, standard deviations, or effect sizes, quantitative meta-analysis was not feasible. Therefore, qualitative synthesis was used to integrate the findings [15].

The qualitative synthesis involved several steps. First, each study’s individual characteristics and main results were organized according to PICO-SD. Data were then classified by intervention type, target group, and outcome category. Studies reporting the same outcome variables were compared for effect trends and consistency, while highly heterogeneous variables were summarized descriptively. This procedure, based on qualitative synthesis guidelines, is appropriate for structuring diverse interventions and results to yield meaningful insights when quantitative integration is not possible [14,15].

## Results

### Literature selection

Based on the literature selection criteria, a total of five studies were included. The selection process was as follows. The literature search identified 17,820 studies: 78 from the domestic database (RISS), 3,595 from PubMed, 12,459 from Embase, 1,211 from CINAHL, and 477 from the Cochrane Library. Of these, 8,284 studies were excluded as duplicates and 2,598 studies were excluded due to lack of access to the original text. Thus, 6,938 studies remained for further screening (Supplementary Table 1).

**Figure 1.**
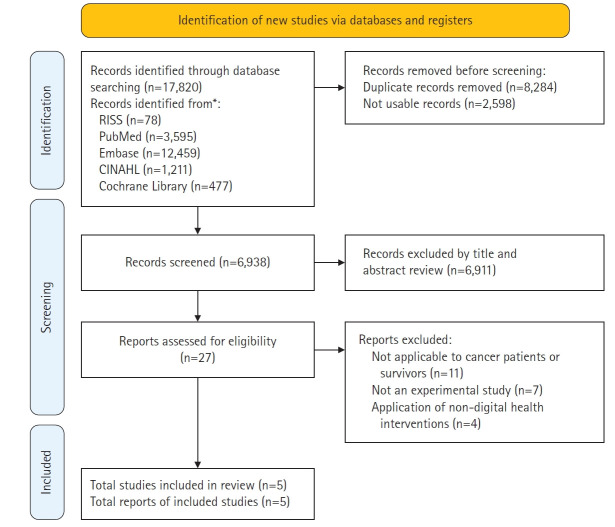
PRISMA 2020 flow diagram of study selection.

Next, 6,911 studies were excluded after reviewing titles and abstracts for non-compliance with the selection criteria, leaving 27 studies for full-text review. Among these, 22 were further excluded: 11 did not target cancer patients or survivors, two were qualitative studies categorized as non-experimental, one was a methodological study, one was a mixed-methods study, three were protocol studies, and four did not apply digital healthcare-based interventions. Ultimately, five studies were included in the review [16-20] (Figure 1).

### Risk of bias assessment of the literature

**Figure 2.**
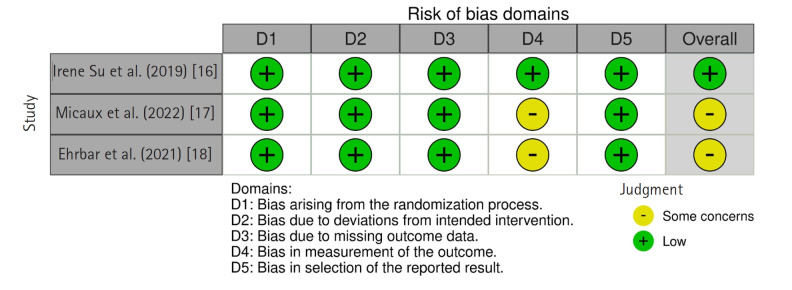
Risk of bias summary of randomized controlled trials using the Cochrane Risk of Bias 2.0 (RoB 2.0) tool.

**Figure 3.**
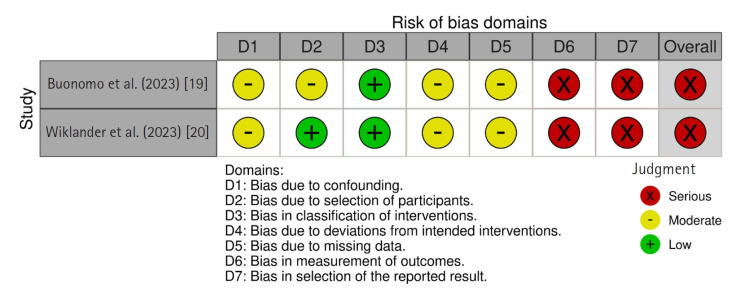
Risk of bias summary of nonrandomized controlled trials using the Risk of Bias in Non-randomized Studies of Interventions (ROBINS-I) tool.

Of the five included studies, three were RCTs assessed using the RoB 2.0 tool [16-18], and two were preliminary studies assessed with the ROBINS-I tool [19,20]. Among the three RCTs, one study was rated as having a low risk of bias [18], while the other two had some concerns [16,17] (Figure 2). Both preliminary studies [19,20] were found to have a serious risk of bias in at least one of the seven domains, but none were rated as having a critical risk of bias. Therefore, in accordance with Cochrane guidelines [21], all studies were included in the analysis (Figure 3).

### Characteristics of the literature

The characteristics of the five intervention studies included in this review are summarized in Table 1, with RCTs listed first and studies arranged by publication year. The publication years ranged from 2017 to 2023, with one study published each year. Sweden contributed two studies (40.0%) [16,20], while Switzerland [17], the United States [18], and Italy [19] each contributed one (20.0%). Most studies (four, 80.0%) [16,17,19,20] targeted cancer patients, while one targeted cancer survivors. Regarding sample size, one study (20.0%) [20] enrolled fewer than 50 participants, two studies (40.0%) [17,19] had 50 to fewer than 100 participants, and two studies (40.0%) [16,18] included 100 or more participants. All five studies (100%) used web-based digital health interventions for oncofertility. The control group was standard care in two studies (40.0%) [16,17], URL transmission in one study (20.0%) [18], and was absent in two studies (40.0%) [20,21]. As for timing, four studies (80.0%) [16-19] assessed outcomes at baseline, one (20.0%) [16] during the intervention, and two (40.0%) [16,17] post-intervention. All five studies (100%) measured effectiveness immediately after the intervention.

### The effect of digital health interventions

The effects of digital health interventions for oncofertility identified in this systematic review were categorized inductively into four domains—symptom management, emotional, cognitive, and usability—based on similarities in the content and characteristics of each study’s outcome variables. The specific effects observed in each domain are summarized in Table 2.

#### Symptom management domain

One study [18] reported on symptom management as an outcome. In this study of 182 participants, 58.5% of the intervention group reported a reduction in hot flashes, 42.5% reported reduced vaginal atrophy, and 50% practiced contraception effectively; however, there were no significant differences between the intervention and control groups for these outcomes. Significant differences between groups were observed for health behavior related to the management of hot flashes and vaginal atrophy, but not for contraception practice.

#### Emotional domain

The effects of digital health interventions for oncofertility in the emotional domain were assessed using several outcomes: fertility stress, anxiety, depression, self-efficacy, decisional regret, and health-related quality of life. Two studies [16,17] evaluated fertility stress in a total of 283 participants. One study [16] found that digital interventions for oncofertility significantly reduced fertility stress immediately after the intervention. In contrast, the other study [17] did not find a statistically significant difference in the overall fertility stress score. However, when examining six subdomains—reproductive potential, sharing with a partner, child’s health, acceptance, pregnancy preparation, and personal health—significant improvements were observed in all areas except for 'child’s health' in the intervention group compared to the control group. Anxiety, depression, and self-efficacy were also assessed in one study [17] with 101 participants, but no significant differences were observed between the intervention and control groups. Decisional regret was analyzed in one study [18] with 51 participants; although the intervention group had lower scores than the control group, the difference was not statistically significant. Health-related quality of life was reported as an outcome in one study [17] with 101 participants, and again, no significant improvement was observed following the intervention.

#### Cognitive domain

The cognitive effects of digital interventions for oncofertility were evaluated using outcomes such as attitude, healthcare facility visits, and knowledge. Two studies [18,19] assessed attitude in a total of 104 participants and found no significant differences between intervention and control groups in either study. Two studies [16,19] investigated healthcare facility visits in 235 participants: one study [16] reported no significant difference between groups, and the other [19] found that only 15.1% of participants visited healthcare facilities. Knowledge was examined in two studies [17,18] with a combined total of 152 participants. Of these, one study [17] showed a significant improvement in knowledge, while the other [18] did not observe a statistically significant difference.

#### Usability domain

Usability was evaluated in terms of the feasibility and acceptability of the digital health interventions’ delivery methods. Four subdomains were used to assess the potential utility of the interventions: demand, acceptability, preliminary efficacy, and functionality. Demand was measured by actual usage indicators such as login frequency, number of completed modules, and forum participation. Acceptability reflected participants’ responses to aspects such as content structure, language level, and the appropriateness of visual materials, and was assessed through online evaluation items and post-intervention interviews. Preliminary efficacy was evaluated based on improvements in knowledge about sexual problems and fertility distress, as well as enhanced coping abilities. Functionality was measured by participants’ experiences with technical performance and user convenience. In one study [20] involving 23 participants, 65.2% demonstrated high demand for the intervention, and overall, participants expressed positive responses regarding acceptability, preliminary efficacy, and functionality.

## Discussion

This study was conducted to analyze research trends in digital health interventions for oncofertility targeting cancer patients and survivors, to comprehensively review their efficacy, and to establish a foundation for future intervention development. The characteristics of the interventions and their efficacy by domain were identified based on the five selected studies.

Since 2017, research on digital health interventions for oncofertility has gradually increased, reflecting a growing interest in fertility preservation and the need for digital health approaches. However, the total number of studies remains limited, and most interventions are restricted to web-based formats. While web-based interventions offer advantages such as accessibility and self-directed learning, they also present several limitations, including information overload, reduced engagement, and challenges in maintaining continuous learning [22,23]. All five studies included in this review utilized web-based elements, but the intervention designs varied. For example, the Fex-Can project [17] was a fully web-based intervention with structured self-learning modules, video materials, and expert Q&A, whereas the insenoallasalute.it project [19] was a hybrid platform combining information provision, screening, and telehealth linkage, making it less of a purely web-based intervention. Such differences in design may influence outcomes related to user engagement, intervention persistence, and emotional or cognitive effects, beyond mere platform accessibility [24].

In the symptom management domain, digital health interventions demonstrated some positive changes—such as improved contraception practices and relief of hot flashes—in certain studies, but consistently significant differences compared to control groups were not observed. The studies included in this review primarily addressed general reproductive health indicators, such as contraceptive use and relief of menopausal symptoms, making it difficult to compare with previous research that emphasized direct symptoms like genital pain and sexual dysfunction [25]. It has been reported that genital pain and sexual dysfunction significantly impact the quality of life in young cancer survivors [26], and cognitive-behavioral therapy-based interventions have been suggested as effective for alleviating these symptoms [23]. These considerations indicate that future digital health interventions for oncofertility should place greater focus on the evaluation of specific genital-related symptoms.

With respect to behavioral change, the current interventions present limitations. Although some studies suggested that self-learning web programs may enhance users’ self-efficacy and health behavior practices, the evaluation of actual behavioral change was limited [20]. Conversely, a chatbot intervention study with pregnant women demonstrated that periodic feedback was effective in sustaining health behaviors [27], implying that future interventions for cancer patients should incorporate tools such as chatbots or notification systems to promote behavior change.

In the emotional domain, some positive effects were observed regarding the reduction of fertility-related stress, but due to limited intervention intensity and duration, significant changes in major psychological outcomes, such as decisional regret, were not detected. The interventions included in this review also tended to have relatively weak emotional support functions, highlighting the need for explicit integration of emotional support elements in future research. In contrast, positive effects on knowledge improvement were consistently reported in the cognitive domain, indicating this as a key strength of digital interventions. However, prior studies suggest that the level of personalization and practical applicability of information has a greater impact on cognitive outcomes than the sheer amount of information provided [22,28,29]. For example, web-based survivorship care plans were shown to improve knowledge and communication skills among young breast cancer patients [17], and decision-support interventions increased patient confidence in their decisions [30,31]. Compared to such studies, some interventions in this review had lower levels of interactivity and personalized information provision, which are important directions for future design improvement.

In terms of usability, high levels of acceptability and accessibility for digital interventions were reported [20]. Particularly when dealing with sensitive topics such as reproductive health, digital platforms can provide anonymity and convenience, potentially lowering access barriers compared to traditional face-to-face counseling [32,33]. However, gaps in acceptability may arise based on users’ technological familiarity and digital literacy [34,35], underscoring the need for complementary designs and support systems that ensure accessibility for users with varying levels of digital competence.

It should also be noted that some studies included in this review were evaluated as having a “serious” risk of bias, which may affect the reliability of the results [15]. It is therefore important to recognize such methodological limitations and to conduct high-quality RCTs in future research. Additionally, there was heterogeneity among the included studies in terms of SD, intervention methods, and evaluation time points, making interpretation of intervention efficacy more challenging [14]. Future research on digital health interventions for oncofertility should strive for consistency in intervention design, analytical strategies that allow comparison of individual components, use of standardized evaluation tools, and incorporation of long-term follow-up.

Despite these limitations, this study has significance in that it provides a comprehensive review of the current status and characteristics of digital health interventions for oncofertility, both domestically and internationally. It offers foundational data for guiding the development of digital health interventions to support reproductive health among cancer patients and survivors and for exploring their practical application.

Future research should expand beyond web-based interventions to develop and evaluate approaches utilizing mobile apps, wearable devices, and virtual reality platforms. It is also necessary to design interventions that integrate personalized information provision tailored to user needs along with emotional support. Interventions should be developed with a focus on behavioral change theories to enhance sustainability and evaluated for long-term effects. Furthermore, key outcome variables for efficacy should be clearly defined, and empirical, structured studies centered on these variables should be accumulated.

## Figures and Tables

**Table 1.** Descriptive summary of included studies (N=5)

First author, year [Ref]	Study design	Country	Sample/population	Sample size	Intervention (program name)	Description	Control group	Outcomes	Timepoints
Micaux et al., 2022 [16]	RCT	Sweden	Breast, cervical, ovarian, testicular cancer, brain tumors, lymphoma patients (19–40 years)	101 (I: 48, C:53)	Web-based (Fex-Can)	12-week web-based psychoeducational program including videos, exercises, texts, and peer vignettes	Standard care	1) Fertility distress	Baseline, 3, 6, and 12 months
								2) Quality of life, knowledge, self-efficacy, anxiety, depression	
Ehrbar et al., 2021 [17]	RCT	Switzerland	Breast cancer, lymphoma patients, others (18–40 years)	51 (I: 24, C: 27)	Web-based (online decision aid)	Online interactive tool including value clarification and tailored information on fertility preservation	Standard counseling	Knowledge, attitude, decisional regret	Baseline, 2 weeks, 6 months
Irene Su et al., 2019 [18]	RCT	United States	Breast cancer survivors (18–45 years)	182 (I: 86, C: 96)	Web-based (WH-SCP)	Web-based personalized survivorship care plan for fertility and reproductive health with e-mail prompts and interactive content	Send the URL	1) Hot flashes, fertility distress, contraception, vaginal atrophy	Baseline, 24 weeks
								2) Healthcare facility visit	
Buonomo et al., 2023 [19]	Quasi-experimental (pre–post)	Italy	Breast cancer patients (not reported)	53	Web-based telehealth (insenoallasalute.it)	Web portal providing fertility info, screening questionnaires, and telehealth support during COVID-19	None	Attitudes, healthcare facility visits	Baseline, post-intervention (timing not specified)
Wiklander et al., 2017 [20]	Feasibility studyz	Sweden	Breast, cervical, ovarian, testicular cancer, CNS tumors, lymphoma patients (18–43 years)	23	Web-based (self-help web-based intervention)	2-month web self-help with videos, exercises, and CBT-based modules for sexual and fertility distress	None	Demand, acceptability, preliminary efficacy, functionality	Post-intervention

C: Control group; CBT: cognitive-behavioral therapy; CNS: central nervous system; COVID-19: coronavirus disease 2019; Fex-Can: fertility and sexuality after cancer; I: intervention group; RCT: randomized controlled trial; Ref: reference; WH-SCP: Women’s Health Survivorship Care Plan.

**Table 2.** Effects of digital health interventions for oncofertility (N=5)

Outcomes	Categories	Reference (direction of effect)
Symptom management domain	Hot flashes	[18] (↔)
	Contraception	[18] (↔)
	Vaginal symptoms	[18] (↔)
	Health behavior practice	[18] (↔)^†^
Affective domain	Fertility distress	[16] (↔)^‡^, [18] (↓)
	Anxiety	[16] (↔)
	Depression	[16] (↔)
	Self-efficacy	[16] (↔)
	Decisional regret	[17] (↔)
	Quality of life	[16] (↔)
Cognitive domain	Attitude	[17] (↔), [19] (↔)
	Healthcare facility visit	[18] (↔), [19] (↔)
	Knowledge	[16] (↑), [17] (↔)
Usability domain	Demand	[20] (↑)
	Acceptability	[20] (↑)
	Preliminary efficacy	[20] (↑)
	Functionality	[20] (↑)

† Although no significant difference was observed in the total score, significant differences between the intervention and control groups were found in subdomains related to the management of hot flashes and vaginal symptoms.

‡ Although no statistically significant difference was found in the total score, significant differences were identified in subdomains such as reproductive potential, sharing with partner, child’s health, acceptance, pregnancy preparation, and personal health.

## Supplementary materials

Further details on supplementary materials are presented online (available at https://doi.org/10.4069/whn.2025.06.13).

Supplementary Table 1. Search strategies
