# Supplementary material for: Digital health interventions for oncofertility in female patients: a systematic review
Source: Womens Health Nurs. 2025 Jun 30;31(2):119–29. doi: 10.4069/whn.2025.06.13 (PMC12245538; doi:10.4069/whn.2025.06.13)
Supplement: Supplementary Table 1. — Search strategies [file whn-2025-06-13-Supplementary-Table-1.pdf]

**Supplementary Table 1.** Search strategies

| Database | Search term |
|---|---|
| Pubmed | (("oncofertility"[All Fields]) OR ("fertility preservation"[MeSH Terms] OR "fertility"[All Fields])) AND ("cancer"[MeSH Terms] OR "neoplasms"[MeSH Terms] OR "cancer"[All Fields]) AND(("mobile applications"[MeSH Terms] OR "mobile"[All Fields]) OR ("web"[All Fields] OR "online"[All Fields]) OR ("application"[All Fields] OR "app*"[All Fields]) OR ("artificial intelligence"[MeSH Terms] OR "AI"[All Fields]) OR ("telemedicine"[MeSH Terms] OR "tele*"[All Fields]) OR ("digital"[All Fields]) OR ("technology"[All Fields])) |
| Embase | ('oncofertility'/exp OR oncofertility OR 'fertility preservation'/exp OR 'fertility preservation' OR fertility) AND ('cancer'/exp OR cancer OR neoplasms) AND |
| | ('mobile application'/exp OR 'mobile app' OR 'mobile application' OR 'mobile applications' OR 'mobile apps' OR 'tablet application' OR 'online app' OR 'online application' OR 'online'/exp OR 'web-based intervention'/exp OR 'internet-based intervention' OR 'internet-intervention' OR 'online-based intervention' OR 'online-intervention' OR 'web intervention' OR 'web-based intervention' OR 'mhealth'/exp OR 'telehealth'/exp OR ehealth OR 'e health' OR 'tele health' OR 'tele*' OR 'AI' OR 'artificial intelligence'/exp OR 'digital' OR 'technology') |
| CINAHL | (oncofertility OR "fertility preservation" OR fertility) AND (cancer) AND |
| | ("mobile" OR "web" OR "online" OR "app*" OR "application" OR "artificial intelligence" OR "AI" OR "tele*" OR "digital" OR "technology") |
| Cochrane | #1 ("oncofertility"):ti,ab,kw OR "fertility preservation":ti,ab,kw OR "fertility":ti,ab,kw |
| Library | #2 MeSH descriptor: [Mobile Applications] explode all trees |
| | #3 MeSH descriptor: [Telemedicine] explode all trees |
| | #4 MeSH descriptor: [Internet-Based Intervention] explode all trees |
| | #5 MeSH descriptor: [Artificial Intelligence] explode all trees |
| | #6 ("mobile" OR "web" OR "online" OR "app*" OR "application" OR "AI" OR "tele*" OR "digital" OR "technology"):ti,ab,kw |
| | #7 #2 OR #3 OR #4 OR #5 OR #6 |
| | #8 MeSH descriptor: [Neoplasms] explode all trees |
| | #9 "cancer":ti,ab,kw |
| | #10 #8 OR #9 |
| | #11 #1 AND #7 AND #10 |
